# Anticipating a new pandemic; what to expect for musculoskeletal extremity injuries at the Emergency Department

**DOI:** 10.1016/j.jorep.2025.100675

**Published:** 2026-02

**Authors:** Dorien A. Salentijn, Thijs H. Geerdink, Ruben N. van Veen, Marit v Barreveld, Marcel G.W. Dijkgraaf

**Affiliations:** a Amsterdam UMC, Department of Epidemiology and Data Science Methodology, Meibergdreef 9, 1105, AZ, Amsterdam, the Netherlands; b OLVG, Department of Trauma Surgery; Amsterdam Public Health, Jan Tooropstraat, 164 1061, AE, Amsterdam, the Netherlands; c Amsterdam Public Health, Meibergdreef 9, 1105, AZ, Amsterdam, the Netherlands

## Abstract

**Background:**

The rapid, extensive spread of COVID-19 had a profound impact on our healthcare system. The imposed restrictions by the governments impacted the incidence of musculoskeletal injuries. Comparative data on number of patients, types of injuries, underlying trauma mechanisms, and proportion of operative treatment in musculoskeletal extremity injuries can be of value to healthcare facilities when preparing for potential future pandemics, providing valuable insights into various crucial aspects of healthcare management, encompassing ED-management, operation room planning, surgical and orthopedic floor management, and out-patient clinic management.

**Methods:**

Count data for this retrospective study was gathered in a large urban teaching hospital and level 2 trauma center in the Netherlands during calendar weeks 11–18 in the pre-pandemic years 2018/2019 and pandemic year 2020 for adults and children. Types of injury were classified into eight distinct anatomic regions: shoulder/upper arm, elbow, lower arm/wrist, hand/finger, knee, lower leg, ankle, and foot.

**Results:**

In the years 2018, 2019, and 2020, a total of 1651, 1877, and 950 patients presented to the ED with musculoskeletal extremity injuries. In comparison to the pre-pandemic years 2018 and 2019 there was a decline of 701 patients (43 % reduction) and 927 patients (49 % reduction). The reduction was greater among children than adults. The decline in sports-related injuries is partially offset by injuries caused by other trauma mechanisms. A significant difference (P = 0.005) was observed for operative treatment in the region of “hand/finger” (7 % vs 13 %).

**Discussion:**

The pandemic resulted in a significant decrease of patients visiting the ED with musculoskeletal extremity injuries. The overall surgical operation rate remains comparable to pre-pandemic years, leading to a considerable reduction in the absolute number of patients requiring surgical treatment. This information can be valuable for hospitals in reallocating healthcare staff to provide essential care for patients in a future pandemic with governmental restrictions.

## 1 Introduction

After a rapid worldwide spread of COVID-19, the World Health Organization declared a pandemic on March 11th, 2020.^1^ The rapid and extensive spread of this disease had a profound impact on our healthcare system. To alleviate the burden on hospitals and their personnel, guidelines for out-patient-clinic management were implemented to establish less face-to-face consultations in orthopedic-trauma management.^2^ In respond, alternative caretaking programs such as Virtual Fracture Clinics (VFC) were swiftly adopted and implemented using video- and telephone consultation in order to minimize hospital visits.^3^

During the pandemic, governments across the globe implemented significant restrictions that had profound consequences on their citizens. In the Netherlands, the COVID-19 lockdown was initiated on March 12, 2020.^3,4^ This lockdown involved the closure of schools, sport facilities, restaurants, and bars, while community centers and other social gathering places were designated as restricted areas. Citizens were asked to limit their outings and prioritize staying within their own homes to minimize physical contact with others. The imposed restrictions reduced the number of new musculoskeletal injuries in the population drastically.^5^ As expected, this affected the demand for hospital care.

Comparative data on the number of patients, types of injuries, underlying trauma mechanisms, and the proportion of operative treatments in musculoskeletal extremity injuries at an ED during a pandemic, as opposed to the pre-pandemic years 2018 and 2019, can be of value to healthcare facilities when preparing for future pandemics. Such data provides valuable insights into various crucial aspects of healthcare management, encompassing ED-management, operation room planning, surgical and orthopedic floor management, and out-patient clinic management. Therefore the aim of this study was to assess the influence of the governmental restrictions during the COVID-19 lockdown in 2020 on musculoskeletal extremity injuries observed at a Dutch ED in comparison to the same time period in pre-pandemic years 2018 and 2019.

## 2 Material and method

### 2.1 Study population and data collection

For this retrospective study, all consecutive patients with musculoskeletal extremity injuries who visited the ED during calendar weeks 11–18 in the years 2018, 2019, and 2020 were included. The study was conducted in the Netherlands at a large urban teaching hospital and level-2-trauma center comprising two distinct locations. Ethical approval was obtained by the institutional review board of our hospital (reference number: WO 22.129).

Patients were categorized into three groups based on the year of their presentation: “pre-pandemic year 2018,”pre-pandemic year 2019”, and “pandemic year 2020”. The data collected from the Electronic Patient files were: date of ED-presentation, age, gender, treatment type (i.e. operative or conservative treatment) and type of injury based on the Diagnosis Treatment Combination (DBC) codes which reflect the diagnosis. The type of injury was classified into eight distinct anatomic regions: shoulder/upper arm, elbow, lower arm/wrist, hand/finger, knee, lower leg, ankle, and foot. Additionally, trauma-mechanisms leading to injuries in the region of 1) the shoulder/upper arm and 2) elbow, were manually extracted from the patients files,

The underlying trauma mechanisms leading to these injuries were categorized into five main groups for children and six for adults. Regarding children; 1) Organized sports (e.g. soccer, basketball, rugby, volleyball, judo, BMX, dancing, gymnastics); 2) Playing outside/playground (e.g. stepping, skating, skateboarding, ditch jumping, swing, slide, climbing frame); 3) Fall from bicycle/tripped on the street; 4) Fall inside the house (e.g. during playing, friendly fight with siblings, fall from couch); 5) Injury (accidently) caused by other (e.g. pulling the arm by elderly, unfriendly fights, violence). For adults; 1) Organized sports (e.g. soccer, basketball, field hockey, rugby); 2) Individual sports high-speed (e.g. speed-cycling, (ice-)skating, athletics, snowboarding, skate-boarding, BMX); 3) Individual sports low-speed (e.g. (kick)boxing, karate, wall-climbing, squash, swimming, fitness, badminton, tightrope walking); 4) Fall outside house (e.g. with two-wheeler, tripped on the street, injured during work); 5) Fall inside house (tripped, fall from stairs, during domestic work); 6) Injury (accidently) caused by other ((vehicle)-accident, (un)friendly fight, alcoholic insult).

### 2.2 Outcomes

The primary outcome focused on the absolute number of consecutive patients presenting to the ED with musculoskeletal extremity injuries, categorized by age group (i.e., children <18 years or adults). The secondary outcomes encompassed the absolute and proportional number of patients categorized into the following groups: 1) age group (i.e., children <18 years or adults); 2) anatomic region; and 3) type of musculoskeletal injury. We also assessed the proportion of patients treated operatively for both adults and children. Additionally, we explored the differences in underlying trauma mechanisms for two regions; 1) shoulder/upper arm and 2) elbow. The choice for these regions was data driven, based on a significant increase in relative numbers of injuries during the lockdown in 2020 compared to pre-pandemic years 2018 and 2019 for children and adults, respectively.

### 2.3 Statistical analysis

Patient characteristics were analyzed using descriptive statistics, as appropriate. Baseline data were reported separately for pre-pandemic years 2018 and 2019 and pandemic year 2020. The differences per type of injury were assessed by comparing the number of patients in pandemic year 2020 to the rounded average number of patients in the pre-pandemic years 2018 and 2019 using the Chi-squared tests or Fisher’s exact test. The same statistics were applied to assess the proportion of operatively treated patients between the average of pre-pandemic years 2018 and 2019 and pandemic year 2020. A *p*-value <0.05 indicated statistical significance. All analyses were performed using SPSS version 29.0. Trauma mechanisms responsible for the reported injuries were displayed graphically.

## 3 Results

### 3.1 Baseline characteristics

In the consecutive calendar weeks 11–18 of the years 2018, 2019, and 2020, a total of 1651, 1877, and 950 patients, respectively, presented to the ED with musculoskeletal extremity injuries. Among these patients, 46 %, 49 % and 47 % were female, and 475 (29 %), 562 (30 %) and 243 (26 %) were children, respectively. The baseline characteristics including age and gender are shown in Table 1.

### 3.2 Primary outcome

In 2020 we observed a decline of 701 (42.5 %), respectively 927 (49.4 %) patients with musculoskeletal injuries compared to pre-pandemic years 2018 and 2019. Among children, the absolute number of musculoskeletal injuries in 2020 had declined by 232 (48.8 %) and 319 (56,8 %) compared to 2018, respectively 2019, while the absolute number among adults had declined by 469 (39.9 %), respectively 608 (46.2 %).

### 3.3 Secondary outcomes

#### 3.3.1 Children

Focusing on anatomic regions comparing pre-pandemic years 2018 and 2019 with pandemic year 2020, it is evident that the absolute number of injuries decreased in all regions during the pandemic (Table 2). Injuries in the elbow region and lower arm/wrist region significantly (*p* = 0.001) increased during the pandemic from respectively 9 %–15 % and 24 %–29 %. Injuries in the hand/finger region, ankle region and foot region significantly (*p* = 0.001) decreased from 21 % to 13 %, 24 %–19 % and 8 %–5 %, respectively.

In the pre-pandemic years, the average overall operation rate for musculoskeletal extremity injuries was 5 %. Specifically, fractures in the regions of “elbow” and “under arm/wrist” were operated on in 12 % and 14 % of cases, respectively, while operations for knee and foot injuries were negligible. During the lockdown in 2020, the overall operation rate remained about 5 %. Among the different regions, fractures in the “lower arm/wrist” region had the highest operation rate, with 16 % of cases undergoing operative treatment. Operative treatment of fractures in the “elbow” region decreased from 12 % in the pre-pandemic years to 5 % in the pandemic year. The proportion of patient treated operatively per anatomic region in 2020 did not differ significantly compared to the rounded average proportion of 2018 and 2019 (Table 3).

#### 3.3.2 Adults

The absolute number of injuries decreased in all anatomic regions during the pandemic year 2020 compared to the pre-pandemic years 2018 and 2019. Relatively, more injuries occurred in the shoulder/ upper arm and lower arm/wrist region. Their share increased respectively from 13 % to 18 % and from 11 % to 15 % during the study period. Relatively, fewer injuries occurred in the region of the ankle, a decrease from 25 % to 18 % (Table 2). Regarding the specific types of injuries as presented in Table 2, relatively more elbow dislocations fractures (8 % versus 23 %), metacarpal fractures (22 % versus 35 %), finger dislocations (10 % versus 16 %) and ankle fractures (32 % versus 47 %) occurred during the pandemic. Injuries that were seen relatively less often during the pandemic compared to 2018 and 2019 were carpal fractures (19 % versus 10 %), phalangeal fractures (42 % versus 34 %) and ankle distortions (57 % versus 44 %).

In the pre-pandemic years 2018 and 2019, the overall operation rate for musculoskeletal extremity injuries was 19 %. Specifically, fractures in the regions of “knee” and “lower leg” were operated on in 33 % and 50 % of cases, respectively, while fractures in the regions of “hand/finger” and “foot” were operated on in 7 % and 3 % of cases, respectively. In the pandemic year 2020, the overall operation rate remained 19 %.There were minor shifts in the percentages of operative treatment observed for most regions (Table 3). A significant difference was observed for operative treatment in the region of “hand/finger” (7 % vs 13 %), with a *p*-value of 0.005.

### 3.4 Exploratory outcomes

**Fig. 1:**
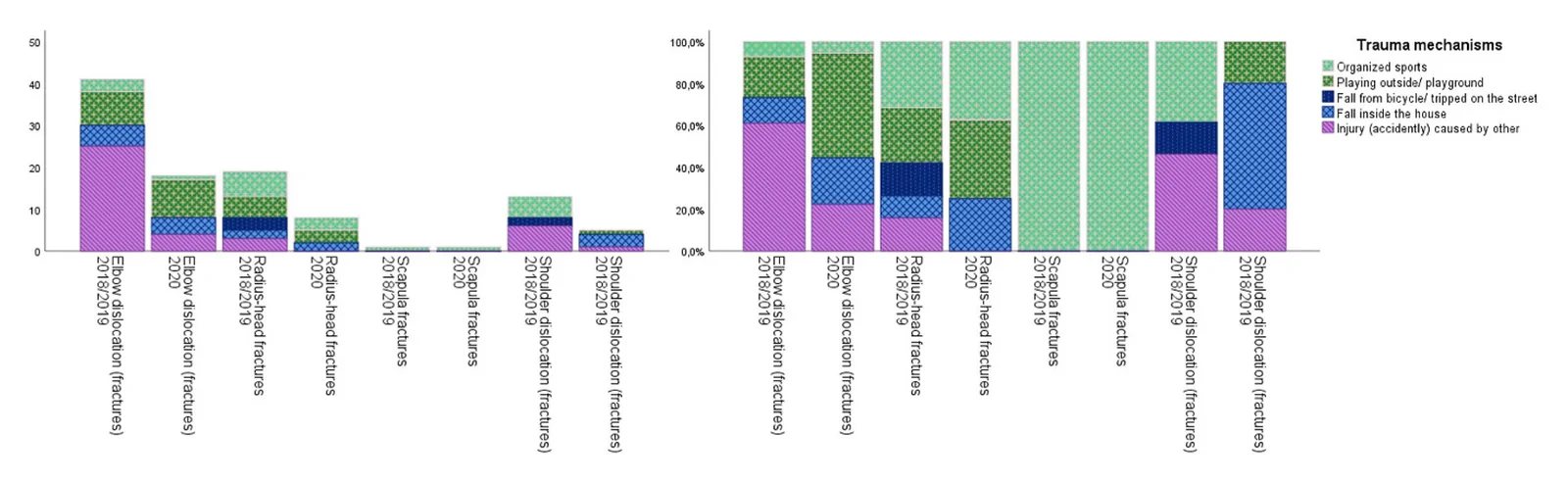
Underlying trauma mechanisms per type of injury per year in children value scaled (left) scaled 100 % (right).

We observed a clear absolute reduction in sports-related injuries for injuries concerning the elbow and shoulder/upper arm regions when comparing the pandemic year 2020 with pre-pandemic years 2018/2019, (Fig. 1). Furthermore, there was a notable increase in injuries related to playing outside/playground during the pandemic year 2020. Lastly, the data showed that a higher proportion of injuries occurred while engaging in injuries occurred inside the house during the lockdown.

#### 3.4.1 Adults

**Fig. 2:**
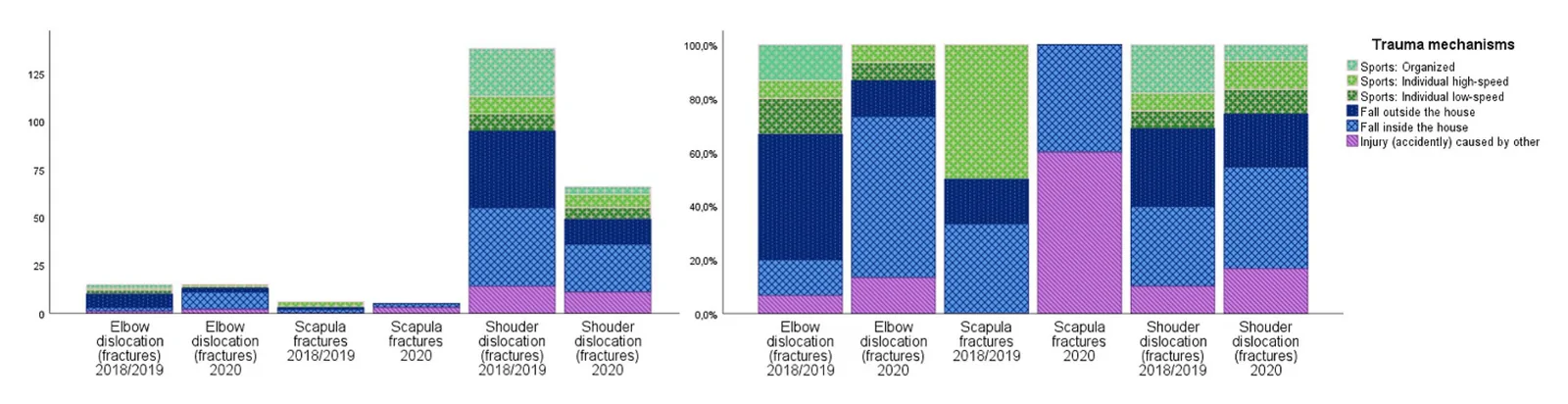
Underlying trauma mechanisms per type of injury per year in adults value scaled (left) scaled 100 % (right).

We observed an overall reduction in injuries caused by sports activities (Fig. 2). Furthermore, injuries resulting from falling outside due to tripping or falling with a regular bicycle notably decreased across all injury types. Conversely, injuries from falls inside the home during activities like domestic work and injuries caused by other objects (e.g., fighting or fingers caught in doors) showed a proportional increase in all types of injuries.

## 4 Discussion

Consistent with what could be anticipated after the implementation of lockdown restrictions, the findings of this study clearly show a notable decrease in the number of patients visiting the ED with musculoskeletal extremity injuries during the initial COVID-19 lockdown in 2020 compared to the corresponding periods in the years 2018 and 2019.

Our hypothesis is that the suspension of sporting activities played a significant role in the observed decline of musculoskeletal extremity injuries. It is well-known that a considerable portion of such injuries are typically associated with sports-related incidents.^5,6^ Supporting this idea, NOC*NSF, the association for organized sports in the Netherlands, conducted an assessment and published clear evidence of a reduction in the number of people participating in sports from March 2020 compared to the same period in 2019. This decline was particularly pronounced among children, which aligns perfectly with the results of our study^6^

The overall assessment in underlying trauma mechanisms for the shoulder/upper arm and elbow region in this study supports the work of NOC*NSF by showing major absolute reductions in organized sports in children and adults. Nevertheless, this reduction in organized sports is (partly)substituted by other activities still leading to injuries. As an example, in adult patients during the year 2020, there was a notable substitution of organized team sports like soccer, field hockey, and rugby with individual non-organized sports characterized by high-speed activities, such as bicycle racing and ice-skating. This substitution to individual high speed sports may have caused more severe injuries leading to the significant higher operation rate during the pandemic in adults with hand fractures (6.5 % vs 13 %).^7–9^ A more detailed study is needed to assess specifically this hand injuries during COVID lockdown. Additionally, individual non-organized sports with low-speed speed, such as (kick)boxing, karate, wall-climbing or swimming, also showed an increase in representation.

The significant reduction in the absolute number of patients presenting to the ED may be beneficial for ED floor management. Having knowledge and data on the expected numbers and types of traumatic extremity injuries during a pandemic is likely to be invaluable for redistributing the ED staff on the floor, thereby improving workflow and optimizing the use of available spaces. For instance, with a 50 % decrease in traumatic injuries requiring casting each day, it becomes possible to reduce cast room staff by 50 % and redeploy them to provide essential care for future pandemic patients.

Furthermore, this data also holds significant implications for the management of orthopedic outpatient clinics. A decreased number of patients needing follow-up visits (both operatively and non-operatively) at the outpatient clinic can alleviate the pressure on the entire clinic floor. For instance, with a major reduction in injuries, nursing staff can efficiently combine desk-work for two orthopedic surgeons simultaneously. As a result, one orthopedic surgeon and one nurse can be reallocated to pandemic care.

During a pandemic, there is a minimal shift in the operation rate per injured region for both adults and children. Overall, children tend to undergo slightly more operations during a pandemic, while for adults, the operation rate decreases only slightly compared to non-pandemic years. Although the overall surgical operation rates during a pandemic are comparable to pre-pandemic years 2018/2019, it is crucial to acknowledge that there are significant implications for OR planning, orthopedic floor management, and outpatient clinic management due to a substantial reduction in the total number of patients undergoing operative treatments. For instance, when comparing the eight weeks of the COVID pandemic lockdown to the pre-pandemic years 2018 and 2019, there was a notable reduction in the number of children treated operatively by 13, while the reduction for adult patients was even more significant at 45. This reduction in operative cases has far-reaching implications across the entire healthcare chain. It leads to reductions in (day-)hospitalizations, children’s nursing care, medicine usage, anesthesia requirements, operating room time, OR staff, surgeons’ workload, and subsequent follow-up at the outpatient clinic. As an example in our hospital, this leads to a weekly reduction of 7.25 patients receiving operative treatment. This translates to more than a full day’s worth of OR time, OR staff, and the combined workload of a trauma surgeon and anesthesiologist for a single workday. This information could prove valuable for future pandemics or lockdown situations, as it highlights the potential for healthcare staff to be partially or fully reassigned to provide essential care for pandemic-affected patients.

This study has several strengths; this research offers a suitable analysis of individuals with musculoskeletal injuries who came to the ED both before and after the pandemic. The study goes a step further by categorizing patients based on eight distinct anatomic regions or specific injury types. The insights derived from this study are valuable in understanding shifts in injury rates, patient attributes, surgical intervention rates, and even the underlying mechanisms of trauma during the stringent lockdown and isolation measures. The implications of these findings are significant, spanning across the management of ED resources, outpatient clinics, and surgical floors.

It is also important to acknowledge the limitations of this study. The retrospective nature of the study restricts the depth of analysis into underlying trauma mechanisms. Furthermore, only 2 regions were assessed for this matter. Subdividing the data into anatomic regions or injury types results in relatively small sample sizes, which can impede a more intricate examination. Our analysis was limited to the calendar weeks that fully encompassed the lockdown phase of the COVID-19 pandemic, being a strength as well a limitation. Nevertheless, within the context of the study’s objectives, these limitations are addressed sufficiently.

## 5 Conclusion

In conclusion, the occurrence of a pandemic resulted in a significant decrease in the total number of patients visiting the ED, which has substantial implications for ED floor management and outpatient clinic management. The decline in sports-related injuries is partially offset by injuries caused by other trauma mechanisms for the two regions studied. Despite the pandemic, the overall surgical operation rate remains comparable to pre-pandemic years, leading to a considerable reduction in the absolute number of patients requiring surgical treatment. This information can be valuable for hospitals in reallocating healthcare staff to provide essential care for patients affected by the pandemic.

## Figures and Tables

**Table 1:** Patient characteristics.

	Children			Adults		
Characteristics	2018 Pre-pandemic n = 475	2019 Pre-pandemic n = 562	2020 Pandemic n = 243	2018 Pre-pandemic n = 1176	2019 Pre-pandemic n = 1315	2020 Pandemic n = 707
Age: median (IQR^a^); extremes	9 (6–13); 0–17	10 (6–13); 0–17	9 (5–12); 0–17	38 (27–58); 18–97	41 (28–60); 18–100	41 (28–60); 18–98
Female: n (%)	186 (39)	245 (44)	110 (45)	544 (46)	638 (49)	333 (47)

a IQR: Interquartile range.

**Table 2:** Absolute and proportional numbers of patients per type of injury per year and per anatomic region per year for children and adults.

		Children						Adults				
*Total Region*		2018/2019		2020		*p*-value^a^		2018/2019		2020		*p*-value^a^
*Injury type*		n = 1037		n = 243				n = 2491		n = 707		
***Shoulder/upper-arm***		***75 (7)***		***24 (10)***		***0.001***		***329 (13)***		***126 (18)***		***<0.001***
Clavicle fracture		41 55)		13 (54)		0.779		73 (22)		26 (21)		0.055
Scapula fracture		1 (1)		1 (4)				6 (2)		5 (4)		
Shoulder-/AC-dislocation		13 (17)		5 (21)				138 (42)		66 (52)		
Humerus fracture proximal/shaft		20 (27)		5 (21)				112 (34)		29 (23)		
***Elbow***		***96 (9)***		***37 (15)***		***0.001***		***192 (8)***		***66 (9)***		**<*0.001***
Distal humerus fracture		31 (32)		10 (27)		0.833		10 (5)		2 (3)		0.012
Elbow dislocation/(fracture)		41 (43)		18 (49)				15 (8)		15 (23)		
Olecranon fracture		5 (5)		1 (3)				26 (14)		8 (12)		
Radius-head fracture		19 (20)		8 (22)				141 (73)		41 (62)		
***Lower arm/wrist***		***245 (24)***		***71 (29)***		***0.001***		***285 (11)***		***103 (15)***		**<*0.001***
Distal radius/antebrachial fracture		245 (100)		71 (100)				285 (100)		103 (100)		
***Hand/finger***		***221 (21)***		***32 (13)***		***0.001***		***634 (26)***		***169 (24)***		**<*0.001***
Carpal fracture		23 (10)		3 (9)		0.272		119 (19)		16 (10)		*<0.001*
Metacarpal fracture		42 (19)		10 (31)				138 (22)		59 (35)		
Phalangeal fracture		122 (55)		18 (56)				269 (42)		57 (34)		
Finger dislocation		29 (13)		1 (3)				65 (10)		27 (16)		
Mallet finger		5 (2)		0 (0)				43 (7)		10 (6)		
***Knee***		***12 (1)***		***4 (2)***		***0.001***		***92 (4)***		***24 (3)***		**<*0.001***
Patella dislocation/(fracture)		10 (83)		2 (50)		0.182		56 (61)		13 (54)		0.551
Tibia plateau fracture		2 (17)		2 (50)				36 (39)		11 (46)		
***Lower leg***		***52 (5)***		***16 (7)***		***0.001***		***38 (2)***		***13 (2)***		**<*0.001***
Tibia/fibula shaft fracture		52 (100)		16 (100)				38 (100)		13 (100)		
***Ankle***		***252 (24)***		***47 (19)***		***0.001***		***615 (25)***		***130 (18)***		**<*0.001***
Ankle fracture		90 (36)		23 (49)		0.029		195 (32)		61 (47)		0.008
Distortion of ankle		159 (63)		23 (49)				352 (57)		57 (44)		
Achilles-tendon rupture		0 (0)		1 (2)				44 (7)		6 (5)		
Talus fracture		3 (1)		0 (0)				24 (4)		6 (5)		
***Foot***		***84 (8)***		***12 (5)***		***0.001***		***306 (12)***		***76 (11)***		**<*0.001***
Calcaneus fracture		1 (1)		1 (8)		0.234		16 (5)		4 (5)		0.743
Tarsal fracture		0 (0)		0 (0)				21 (7)		6 (8)		
Metatarsal fracture		38 (45)		6 (50)				131 (43)		37 (49)		
Phalangeal fracture		45 (54)		5 (42)				138 (45)		29 (38)		

a Pearson Chi Square.

**Table 3:** The percentage of operative versus non-operative treated patients per anatomic region per year in children and adults.

		Children								*Adults*						
*Region*		2018		2019		2020		p-value^a^		2018		2019		2020		*p*-value^a^
		n = 475		n = 562		n = 243				n = 1176		n = 1315		n = 707		
		n (%)								*n (%)*						
***Shoulder/upper arm***		***41***		***34***		***24***		***0.570*** ^b^		***158***		***171***		***126***		***0.339***
Non-operative		40 (98)		33 (97)		23 (96)				139 (88)		148 (87)		114 (90)		
Operative		1 (2)		1 (3)		1 (4)				19 (12)		23 (13)		12 (10)		
***Elbow***		***47***		***49***		***37***		***0.515*** ^b^		***83***		***109***		***66***		***0.607***
Non-operative		42 (89)		43 (88)		35 (95)				70 (84)		95 (16)		55 (83)		
Operative		5 (11)		6 (12)		2 (5)				13 (16)		14(13)		11 (17)		
***Lower arm/wrist***		***105***		***140***		***71***		***0.665***		***127***		***158***		***103***		***0.548***
Non-operative		91 (87)		212 (86)		60 (84)				100 (79)		124 (78)		78 (76)		
Operative		14 (13)		19 (14)		11 (15)				27 (21)		34 (22)		25 (24)		
***Hand/finger***		***102***		***119***		***32***		***1.000*** ^b^		***311***		***323***		***169***		***0.005***
Non-operative		99 (97)		115 (97)		31 (97)				292 (94)		301 (93)		147 (87)		
Operative		3 (3)		4 (3)		1 (3)				19 (6)		22 (7)		22 (13)		
***Knee***		**5**		**7**		**4**		***-***		***49***		***43***		***24***		***0.747***
Non-operative		5 (100)		7 (100)		4 (100)				33 (67)		29 (67)		17 (71)		
Operative		0 (0)		0 (0)		0 (0)				16 (33)		14 (33)		7 (29)		
***Lower leg***		***17***		***35***		***16***		***0.559*** ^b^		***19***		***19***		***13***		***0.472***
Non-operative		16 (94)		34 (97)		15 (94)				8 (42)		11 (58)		8 (62)		
Operative		1 (6)		1 (3)		1 (6)				11 (58)		8 (42)		5 (38)		
***Ankle***		**120**		**132**		**47**		**0.621** ^b^		***289***		***326***		***130***		***0.165***
Non-operative		117 (98)		129 (98)		45 (96)				255 (88)		292 (90)		110 (85)		
Operative		3 (3)		3 (2)		2 (4)				34 (12)		34 (10)		20 (15)		
***Foot***		**38**		**46**		**12**		**-**		***140***		***166***		***76***		***0.695*** ^b^
Non-operative		38 (100)		46 (100)		12 (100)				137 (98)		161 (97)		75 (99)		
Operative		0 (0)		0 (0)		0 (0)				3 (2)		5 (3)		1 (1)		

a P-value of proportional difference in operative versus non-operative treatment between pre-pandemic years (2018 + 2019) and pandemic year 2020.

b Fisher’s exact. Others chi square.

## Data Availability

Upon reasonable request to the corresponding author.
